# A protocol for developing a core outcome set for ectopic pregnancy

**DOI:** 10.1186/s13063-021-05772-x

**Published:** 2021-11-17

**Authors:** Krystle Y. Chong, Sarah Solangon, James Kemper, Kurt Barnhart, Pamela Causa Andrieu, Perrine Capmas, Carolina Chacon, George Condous, Liesl de Waard, James M. N. Duffy, Andrew Horne, Maria Memtsa, Femke Mol, Munira Oza, Annika Strandell, Madelon van Wely, Janneke van’t Hooft, Lan N. Vuong, Jian Zhang, Davor Jurkovic, Ben W. Mol, International Collaboration on Core Outcomes in Ectopic Pregnancy

**Affiliations:** 1 Monash Women’s, Monash Health, Clayton, Australia; 2 Department of Obstetrics and Gynaecology, Monash University, Clayton, Australia; 3 Institute for Women’s Health, University College London, London, UK; 4 Department of Obstetrics and Gynecology, Division of Reproductive Endocrinology & Infertility, Penn Medicine/University of Pennsylvania School of Medicine, Philadelphia, PA USA; 5 Department of Radiology, Memorial Sloan Kettering Cancer Center, New York, USA; 6 Radiology Service, Hospital Italiano de Buenos Aires, Ciudad Autonoma de Buenos Aires, Argentina; 7 Department of obstetrics and Gynecology, Bicetre University Hospital, Paris Saclay University, Le Kremlin Bicêtre, France; 8 Acute Gynaecology, Early Pregnancy and Advanced Endosurgery Unit, Sydney Medical School Nepean, University of Sydney, Nepean Hospital, Camperdown, NSW Australia; 9 Department of Obstetrics and Gynaecology, Faculty of Medicine and Health Sciences, Stellenbosch University, Cape Town, South Africa; 10 King’s Fertility, Fetal Medicine Research Institute, London, UK; 11 MRC Centre for Reproductive Health, University of Edinburgh, Edinburgh, EH16 4TJ UK; 12 Center for Reproductive Medicine, Amsterdam Reproduction & Development Research Institute, Amsterdam, UMC Netherlands; 13 The Ectopic Pregnancy Trust, London, UK; 14 Department of Obstetrics and Gynecology, Institute of Clinical Sciences, Sahlgrenska Academy, University of Gothenburg, Gothenburg, Sweden; 15 Department of Obstetrics and Gynaecology, Amsterdam UMC, Amsterdam, The Netherlands; 16 Department of Obstetrics and Gynecology, University of Medicine and Pharmacy, Ho Chi Minh City, Vietnam; 17 Department of Obstetrics and Gynecology, International Peace Maternity and Child Health Hospital, School of Medicine, Shanghai Jiaotong University, Shanghai, China

**Keywords:** Ectopic pregnancy, Consensus study, Modified Delphi method, Core outcome set, Randomised controlled trials

## Abstract

**Background:**

Randomised controlled trials (RCTs) evaluating ectopic pregnancy have reported many different outcomes, which are themselves often defined and measured in distinct ways. This level of variation results in an inability to compare results of individual RCTs. The development of a core outcome set to ensure outcomes important to key stakeholders are collected consistently will guide future research in ectopic pregnancy.

**Study aim:**

To develop and implement a core outcome set to guide future research in ectopic pregnancy.

**Methods and analysis:**

We have established an international steering group of key stakeholders, including healthcare professionals, researchers, and individuals with lived experience of ectopic pregnancy. We will identify potential outcomes from ectopic pregnancy from a comprehensive literature review of published randomised controlled trials. We will then utilise a modified Delphi method to prioritise outcomes. Subsequently, key stakeholders will be invited to score potential core outcomes on a nine-point Likert scale, ranging from 1 (not important) to 9 (critical). Repeated reflection and rescoring should promote whole and individual stakeholder group convergence towards consensus ‘core’ outcomes. We will also establish standardised definitions and recommend high-quality measurements for individual core outcomes.

**Trial registration:**

COMET 1492. Registered in November 2019.

## Introduction

Ectopic pregnancy (EP) occurs when the developing embryo implants in a site other than the uterine cavity endometrium and is a potentially life-threatening complication in first trimester pregnancy [1]. It is estimated to affect 1–2% of pregnancies and accounts for 75% of early pregnancy mortality, 6% of direct obstetric causes of mortality in Australia, and up to 8% of maternal deaths globally [2–4]

It has the potential to affect patients not only in the acute setting but also has lasting impacts on future fertility, with only 50% women successfully having a live birth following an ectopic pregnancy [5]. The main categories of treatment fall into expectant management, medical management with methotrexate, or surgical management with a salpingostomy or salpingectomy. The approach towards counselling women with ectopic pregnancies depends not only on the woman’s clinical state, her wishes and level of compliance with treatment, but also her serum beta-hCG, ultrasonography findings and operator experience laparoscopically [1]. Clinically unstable patients with pain and bleeding may require immediate surgical management prior to formal ultrasonography.

Diagnosis has improved significantly in the last few decades, and randomised controlled trials (RCTs) remain the mainstay of assessing the effectiveness and safety of treatments. The pooling of individual RCT data is likely to provide the best evidence to inform clinical practice [6]. However, currently published data in ectopic pregnancy has reported many different outcomes of which many are defined and measured in diverse ways.

An analysis of the published literature in ectopic pregnancy found 30 RCTs primarily studying ectopic pregnancies [7]. Overall, the commonest outcome, treatment success by resolution of ectopic pregnancy was measured both with ultrasonography and serum beta-HCG levels, which used 6 different outcome measurements, ranging from <2 to 20 IU/L.

The lack of consensus and level of variation results in an inability to compare the results of individual RCTs and consequently directly impacts the usefulness of research to inform patients and clinicians in clinical practice. The development of core outcome sets allows for standardisation in outcome reporting and ultimately allows healthcare consumers to make more informed healthcare decisions in a collaborative manner with healthcare professionals.

We propose the development of a core outcome set to ensure outcomes important to key stakeholders are collected consistently, to guide future research in definite and probable ectopic pregnancy.

There is a notable difference in sonographic criteria for the diagnosis of ectopic pregnancy internationally. In literature originating from the USA, the presence of an extrauterine gestational sac with a yolk sac or embryo is required for the formal diagnosis of an EP [8]. This is compared with literature from Europe, UK and Australia, where EP is diagnosed based on the presence of an extrauterine inhomogenous mass known as the ‘blob sign’, or an extrauterine empty gestational sac, characterised by a hyperechoic ring known as the ‘bagel sign’ ([9,10]).

This core outcome set does not seek to reach consensus regarding the diagnostic or management dilemmas surrounding ectopic pregnancy and pregnancy of unknown location with suspected ectopic pregnancy. We also recognise that there are significant variations in ultrasound practice worldwide and the criteria to diagnose ectopic pregnancy are not consistently applied. In addition, ectopic pregnancy is often diagnosed based on biochemical algorithms without the pregnancy being positively identified on the scan. The purpose of this exercise is not to standardise the criteria for diagnosing ectopic pregnancy, but to provide a set of core outcome for reporting the result of interventional studies on the management of ectopic pregnancy regardless of the diagnostic criteria applied in the individual trials.

Core outcome sets are a minimum collection of data sets that are distinct, discriminatory and feasible outcomes which are routinely collected and reported in RCTs. These core outcomes would be standardised with measurement and reporting, allowing key stakeholders including individuals with lived experience, healthcare professionals and research clinicians to compare, combine and contrast results of trials [11].

### Primary outcome

To develop a core outcome set in ectopic pregnancy.

## Materials and methods

**Fig. 1:**
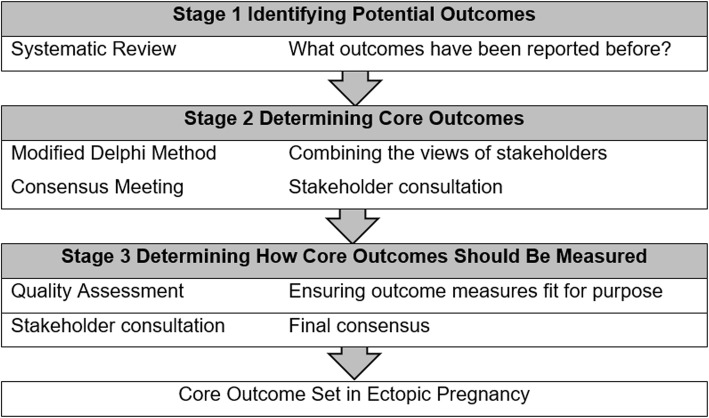
Methodology in development of core outcome sets

The methods have been informed by the Core Outcome Measures in Effectiveness Trials (COMET) Initiative Handbook and other core outcome set development studies relevant to women’s health. This study protocol is reported in accordance with established methodology in development of core outcome sets (Fig. 1).

### Prospective registration

This study has been prospectively registered with the Core Outcome Measures in Effectiveness Trials (COMET) initiative; the registration number is 1492 and is available online (www.comet-initiative.org/studies/details/1492).

### Steering group

An international steering group of 25 individuals, including healthcare professionals, research clinicians and individuals with lived experience with ectopic pregnancies. This steering group has been formed through existing research networks to guide the development of this core outcome set and is composed of clinicians identifying as both research clinicians and healthcare professionals, as well as several individuals with lived experience with ectopic pregnancies who are part of patient advocacy groups.

### Scope of this core outcome set

The steering group recommends that the core outcome set should apply to RCTs, systematic reviews and clinical practice guidelines evaluating interventions for women with ectopic pregnancies.

### Identifying potential core outcomes

Potential core outcomes have been identified through a comprehensive literature review, extracting outcomes in published trials in management of ectopic pregnancy. A literature search was performed utilising PubMed with search terms ‘ectopic pregnancy’, filtered for RCTs published from 1997 to 2017. This search was filtered for studies in humans only and English language publications [7].

This initial search listed 89 studies, 30 of which were excluded for not primarily studying ectopic pregnancy and a further 25 excluded for not meeting CONSORT criteria for RCT. Four full-text studies could not be accessed during the literature review and were hence also excluded. Hence, 30 studies from 1997 to 2017 were analysed.

A total of 30 RCTs reported 13 different measurable outcomes in 4 different domains (Table 1). The commonest outcome, treatment success by resolution of ectopic pregnancy was listed in 76% (23/30) RCTs. However, it was measured with ultrasonography and with serum beta-HCG levels using 6 different outcome measurements, ranging from endpoint bHCG <2 to <20 IU/L.

**Table 1:** Outcomes in ectopic pregnancy RCTs

	Outcome	Studies listed *N*=30 (%)
Resolution	Resolution of ectopic pregnancy	23 (76%)
	βHCG as marker of resolution	22 (73%)
	Ultrasound as marker of resolution	3 (10%)
	Tracking duration listed	12 (40%)
	Repeat intervention—medical or surgical	8 (26%)
	Resolution of clinical symptoms	5 (16%)
Complications	Haemorrhage—intra-operative or post-operative	7 (23%)
	Infection	4 (13%)
	Other surgical complications	3 (10%)
	Pain	3 (10%)
	Medication adverse effects for medical management	9 (30%)
Fertility	Spontaneous pregnancy	8 (26%)
	Conception via artificial reproductive technology	3 (10%)
	Subsequent ectopic pregnancy	1 (3%)
Patient experience	Treatment satisfaction	4 (13%)
	Length of stay	8 (26%)
	Economic impact—cost of care received	1 (3%)
	Psychological impact	1 (3%)

Less commonly listed outcomes included complications of medical or surgical management, fertility and patient experience.

The primary outcome of the RCT was listed in 73% (22/30) studies. Resolution of ectopic pregnancy was the primary outcome in 15 studies, followed by spontaneous conception in 3 studies, and treatment satisfaction in 2 studies. The remainder 2 studies analysed post-operative complications and surgical difficulty.

In addition, we will develop a comprehensive inventory of outcomes in consultation with key stakeholders including healthcare professionals, researchers and individuals with lived experience with ectopic pregnancies. The inventory will then be entered into a modified Delphi method, delivered through sequential online surveys using the Delphi survey software.

### Selecting core outcomes

Using the modified Delphi method, 3 key stakeholder groups including healthcare professionals, research clinicians and individuals with lived experience in ectopic pregnancy will be invited to participate and grade potential core outcomes. Participants are recruited widely across existing research networks and patient advocacy groups, and once consented to participate in the study, all stakeholders will receive email invitations, with a link to access the online Delphi study. Key stakeholders will rank outcomes from one (not important for decision making) to nine (critical for decision making), as well as suggesting further outcomes for study. This series of repeated surveys to key stakeholders allows for assessment of the extent of agreement and disagreement by facilitating repeated reflection and rescoring [12]. Furthermore, web-based scoring systems allow not only for a global level of participation, but also for ranking with anonymity, efficiency and feasibility. There are currently no clear recommendations for calculating the required sample size. Based on previous studies, we will aim to include a minimum of 16 participants from each of the key stakeholder groups [12–14].

### Delphi study pilot

A Delphi survey pilot will be developed using the Delphi Manager software to ensure feasibility and ease of completion for stakeholders using the appropriate and patient-friendly terminology. This Delphi study will be piloted by the study committee and a sample of stakeholders prior to being accessible to all stakeholders. This initial steering group was formed via email invitation through existing research networks.

### Round 1

During round one of the Delphi survey, all participants will be invited to provide their demographic details and will be provided with a unique identifier to facilitate responses to future rounds with anonymity. Outcomes will be listed in individual domains, and key stakeholders will be asked to score individual outcomes using the nine-point Likert scale, which was created by the Grading of Recommendations, Assessment, Development and Evaluation (GRADE) working group, and widely adopted by developers of core outcome sets [15]. Additionally, participants are invited to suggest additional outcomes before completing the first-round survey which will have a 4-week window for completion. All outcomes are summarised as a whole and for individual stakeholder groups using Delphi Manager. Any additional outcomes will be considered by the steering group and potentially included for review in round two.

### Round 2

All outcomes from round one will be then carried forward to round two and key stakeholders will receive individual stakeholder as well as group responses. Participants will be asked to reflect upon any similarities or differences between groups and then score each outcome again, as well as any additional outcomes suggested in round one, using the Likert scale. Following round two, each outcome will be summarised by whole and individual stakeholder responses. A standardised definition will be applied to results to identify core outcomes, as defined by the 70/15% consensus definition as advocated by the COMET initiative [16]. A consensus outcome would be identified when over 70% participants score the potential outcome as ‘critical for decision making’ (with a score of 7–9), and less than 15% of participants scored the outcome as being ‘of limited importance for decision making’ (with a score of 1–3).

### Stakeholder consultation

A final consensus meeting with the core steering group will review the results and aim to develop a final core outcome set for ectopic pregnancy. This meeting will ensure inclusion and fair representation from all stakeholder groups to facilitate an unbiased consensus. If necessary, the steering group may suggest the need for a further Delphi study round. Given restrictions with the COVID-19 pandemic, the steering group may meet for the final consensus meeting via teleconference.

### Standardising core outcome measure

Once a final core outcome set has been selected, the steering group will then determine how these outcomes will be measured. High-quality outcome measures should be sought for each core outcome. Potential definitions of each core outcome will be assessed in consultation with national and international guidelines, then entered into a consensus development workshop involving key stakeholders. The objective of the consensus workshop will be to identify and standardise definitions for individual core outcomes. Potential measurement instruments will be inventoried across systematic reviews, RCTs, and national and international guidelines.

### Ethical review

This study has Monash Health ethics approval as a low-risk study (HREC RES-20-0000099L). All participants involved will be asked for consent prior to registration and participation in the Delphi study. All data collected as part of the Delphi study will be held on secure servers for 5 years.

### Dissemination

Finalising a core outcome set in ectopic pregnancies could help guide and advance future clinical guidelines, RCTs and systematic reviews. Its implementation could ensure that outcomes important to individuals with lived experience, researchers and healthcare professionals are collected in a standardised fashion, to guide and inform clinical practice in the future.

The Standard Protocol Items: Recommendations for Interventional Trials (SPIRIT) statement recommends the use of core outcome sets where they exist [17].

The Core Outcomes in Women’s and Newborn Health (CROWN) initiative, a collection of 80 specialty journals has been established to support, disseminate and implement core outcome sets. Participating journals will require all research clinicians to report core outcomes and outcome measures [18, 19].

## Discussion

A comprehensive literature review found 30 RCTs reported 13 different measurable outcomes in 4 different domains. The commonest outcome, treatment success by resolution of ectopic pregnancy was measured both clinically and with serum beta-HCG levels using 6 different outcome measurements.

This variation in outcome collection has been observed not only in ectopic pregnancy, but also in other areas of obstetrics and gynaecology including pre-eclampsia, preterm birth, endometriosis and polycystic ovarian syndrome ([13, 14, 20, 21]. The development of core outcome sets in these areas has allowed for standardisation in outcome reporting and ultimately allows healthcare consumers to make more informed healthcare decisions in a collaborative manner with healthcare professionals.

### Trial status

This protocol is version number 6, dated on February 21, 2021, recruitment began on March 1, 2021, and all three rounds are anticipated to be completed in July 2021.

## Conclusion

Implementation of a core outcome set will enable collection and reporting of data in a standardised fashion, with data sets important to key stakeholders including individuals with lived experience with ectopic pregnancy. This will allow standardisation of research to guide and inform clinical practice and enhance patient-centred care.

## Data Availability

DelphiManager software, developed and maintained by the COMET Initiative, will be used to undertake the e-Delphi survey. Final dataset will be held on DelphiManager servers, hosted, and provided by University of Liverpool.
